# VOC Emission Analysis of Bitumen Using Proton-Transfer Reaction Time-Of-Flight Mass Spectrometry

**DOI:** 10.3390/ma13173659

**Published:** 2020-08-19

**Authors:** Jaffer Bressan Borinelli, Johan Blom, Miguel Portillo-Estrada, Patricia Kara De Maeijer, Wim Van den bergh, Cedric Vuye

**Affiliations:** 1 Road Engineering Research Section (RERS), EMIB, Faculty of Applied Engineering, University of Antwerp, 2020 Antwerp, Belgium; johan.blom@uantwerpen.be (J.B.); patricija.karademaeijer@uantwerpen.be (P.K.D.M.); wim.vandenbergh@uantwerpen.be (W.V.d.b.); cedric.vuye@uantwerpen.be (C.V.); 2 Research Group PLECO (Plants and Ecosystems), Faculty of Science, University of Antwerp, 2610 Wilrijk, Belgium; miguel.portilloestrada@uantwerpen.be

**Keywords:** volatile organic compounds (VOCs), crumb rubber modified bitumen (CRMB), bitumen fumes, proton-transfer reaction time-of-flight mass spectrometry (PTR-TOF-MS)

## Abstract

Bitumen is one of the most important materials used in roads. During asphalt pavement construction, workers can be affected by emissions, such as volatile organic compounds (VOCs), when bitumen is heated. Therefore, it is crucial to correctly identify and measure VOCs. This paper presents a novel, promising method to determine VOC emissions. The proposed method offers a way to standardize routine measurements on a lab scale, enabling reliable comparison across bitumen types and their modifications or additives. A proton-transfer reaction time-of-flight mass spectrometer (PTR-TOF-MS) was used to monitor VOC emissions from commercial unmodified bitumen and crumb rubber modified bitumen (CRMB) with heating of up to 180 °C. Results confirmed that the temperature range of 160–180 °C is a highly influential factor for VOC emissions from heated commercial bitumen and particularly CRMB. A significant increase in alkane and aromatic emission was detected when the binders were heated to 180 °C. Sulfur-containing VOCs were almost nonexistent for the base bitumen fumes, while a significant increase was detected in the fumes when two different types of CR were added to the bitumen, even at 120 °C. The additional CR in the bituminous binder contributed to the potentially harmful VOC emission of benzothiazole, which belongs to the class of sulfur-containing compounds. The concentration of benzothiazole was 65%, 38%, and 35% higher for CR1 in comparison to CR2 at 140, 160, and 180 °C, respectively. It is clear from the results that this method allows different bitumen sources or modifications to be quickly analyzed and their VOC emissions cross-compared. If adopted and confirmed further, the method could offer the asphalt industry a viable solution to monitor VOC emissions by analyzing samples in real time at different steps of the production process.

## 1. Introduction

Bitumen is a residue of crude oil refinery, which passes through a refinery process to be used as a binder. Although most lightweight compounds in crude oil are removed during this process, there is still some remaining in the final bitumen residue, which can be released as emissions during the production process of asphalt mixture at a high temperature (>150 °C) [1,2]. The most significant components of bitumen fumes, released at higher or lower concentrations, are volatile organic compounds (VOCs), polycyclic aromatic hydrocarbons (PAHs), sulfur, nitrogen oxides, particulates, and carbon monoxide [3,4,5]. These components can also be found in crumb rubber modified bitumen (CRMB). In recent years, new-end market solutions have been developed to find an alternative solution for end-of-life tires (ELTs) than sending them to landfills or burning them [6]. The rubber employed for the fabrication of tires is of high quality and resilient, leading to great potential for high-end recycling. The application of rubberized bitumen for road construction using CR from ELTs has potential in Europe due to governmental support for recycling and increasing concern about waste accumulation. CR blended into bitumen can improve asphalt pavement performance, such as high-temperature stability, fatigue life, and cracking resistance, and can be considered as a possible solution for waste tire recycling [6,7,8,9,10].

During asphalt pavement construction, the bitumen is heated to 160–180 °C. The surrounding air quality is affected by these fumes, which potentially causes harmful health effects to on-site workers [11] due to their toxic and carcinogenic effects [5]. The most common symptoms experienced by workers during short-term exposure (15 min) to high levels of bitumen fumes are irritation of the nose, upper respiratory tract, skin, and eyes. However, these symptoms are reversible once the exposure has stopped [3,11]. VOC concentrations while paving are highly dependent on air movement. Therefore, higher concentrations are registered in the absence of wind. Despite that, the local concentrations of harmful VOCs are usually kept below the exposure limits according to several regulatory agencies [2,11,12].

In Europe, the VOC Solvents Emissions Directive [13] is considered the main policy instrument to reduce VOC emissions. The Chemical Agents Directive (98/24/EC) [14] and the Directive on Carcinogens and Mutagens at Work (2004/37/EC) [15] aim to protect workers from chemical risks at the workplace. The National Institute for Occupational Safety and Health (NIOSH) has evaluated scientific evidence concerning the potential health effects of occupational exposure to bitumen [16]. NIOSH recommended bitumen fumes to be considered as a potential occupational carcinogen based on the results from animal studies. Although carcinogenic PAHs have been identified in bitumen fumes at various worksites, the measured concentrations and the frequency of their occurrence have been low. In order to minimize possible acute or chronic health effects from exposure to bitumen, bitumen fumes, and vapors, a maximum emission of 5 mg/m^3^ during any 15 min period was recommended as a limit [17]. In the study by Lin et al. [5], the concentrations of total VOC and the 11 detected VOC compounds were extremely low and barely exceeded 3 parts per billion (ppb) by volume when tested by a gas chromatograph/mass spectrometer (GC/MS). The VOC concentration was shown to vary in accordance with the type of bituminous mixture and temperature [5]. The temperature is an essential factor when it comes to the amount of VOC emissions, following an exponentially rising curve with the increase in temperature. The bitumen source significantly affects VOC due to the presence of additives added by bitumen suppliers [2]. Moreover, the addition of CR to bitumen contributes to the total VOC emissions and especially the emission of pollutants such as xylene, benzothiazole, and toluene [2]. Due to the significant effect of pollutants in fumes to human health and the complexity of emission dynamics, which depends on the initial composition and the additives used, more insight into the relationship between temperature and VOC emissions is needed, especially if CR is added.

Warm mixture asphalt (WMA) plays an important role in asphalt technology as this technique allows the production of healthier working conditions and environmentally friendly bituminous mixtures by reducing the mixing, production, and compaction temperatures using additives or foamed bitumen. The reduction in temperature for WMA can reach up to 90 °C below that used for hot mixture asphalt [18]. Comparing the emissions from recycled tire rubber modified bitumen in hot and warm mix conditions, the temperature is an important factor when it comes to the emission of VOCs [2]. Using zeolite materials through bitumen foaming can reduce the production and compaction temperatures by 30 °C [19]. Another recent technology is the use of geopolymer additives in WMA. Due to the porous structure of geopolymers, a high capability of absorbing bitumen VOCs is achieved during asphalt production, thereby reducing VOC emissions [20].

Previous studies have included tests performed to simulate the fume generation process using bitumen fume generators in a laboratory [21] and to collect the fumes on-site [2,11,12]. As shown in previous studies [4,22,23], the environment in the field is always different from one site to another. Differences in air temperature, wind speed, sun exposure, materials, mixture and compaction temperatures, sampling methods, or even the combination of all these factors are the reasons for the high discrepancies in the chemical composition and toxicological properties of bitumen fumes that are found between samples collected in the field and those produced in laboratories [11]. Current research methods on laboratory scale include flame ionization detection (FID) and photo ionization detection (PID), which are commonly used to test the total mixed VOCs [24]. In addition, GC/MS and ambient volatile organic canister sampling (AVOCS) are used to determine the VOC components [25]. However, these available measurement methods are selective in what they can accurately measure and quantify [26]. Proton-transfer reaction time-of-flight mass spectrometry (PTR-TOF-MS) represents a useful and innovative solution in the monitoring of VOC emissions. The technique has been successfully applied to environmental research such as measurement of ecosystem VOC fluxes by coupling the instrument to a flux tower [27], to plant VOC emissions by coupling it to a leaf chamber [28], and to air pollution cleaning potential of bacteria by measuring the headspace of the cultures [29]. Other applications include the measurement of VOCs that characterize food flavor [30], indoor air quality studies [31], and breath analysis in medical research [32]. Accordingly, PTR-TOF-MS has potential application for bitumen research and the asphalt sector in monitoring VOC emissions at different steps of the production process [33].

The current study was carried out on a laboratory scale and presents a promising method to identify VOC emission for bituminous samples. This way, different bitumen modifications can be quickly analyzed, and their VOC emissions crossed-compared. In the present study, this method was not used to characterize airborne emissions during on-site work. In fact, it targeted the emission source in controlled conditions to improve the identification of VOCs and, consequently, identify potential health risks for workers before or during pavement construction. Using PTR-TOF-MS, the VOC blend in the fumes of commercial unmodified bitumen was analyzed qualitatively and quantitatively along a gradient of temperatures while being heated. The tests were repeated with the same bitumen containing CR additions of different particle sizes.

The rest of the paper is structured as follows: introduction of the research materials and methods, presentation of the VOC measurements, reporting of the results using a semiquantitative approach, and presentation of the conclusions.

## 2. Materials and Methods

### 2.1. Materials

**Figure 1:**
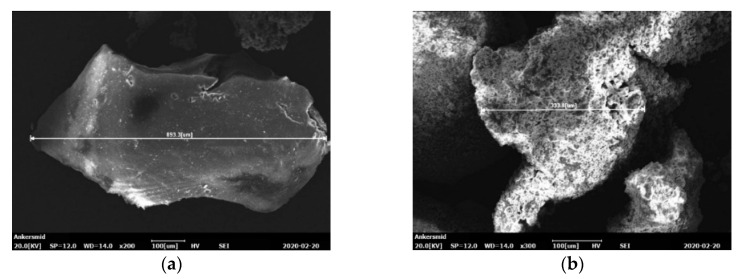
Scanning electron microscopy images of the crumb rubber (CR) particles: (**a**) CR1 and (**b**) CR2.

The base bitumen used in the current study was a standard 50/70 unmodified binder. The physical properties of the bitumen are given in Table 1. The penetration and softening points were obtained in the laboratory following the standards EN 1426 and EN1427, while the density at 25 °C was reported by the supplier. Two different commercially available crumb rubber types (named CR1 and CR2) were used in the current study as modifications of the base bitumen. Both CR were produced by ambient grinding (i.e., passenger car tires milled into small particles at ambient temperature). This type of grinding generates irregularly shaped particles with relatively large surface areas, which improves the interaction between bitumen and CR [10]. This type of grinding is also the most used and the most cost-effective way to process ELTs. CR1 particles have an approximate length of up to 0.8 mm (Figure 1a), while CR2 is composed of smaller-sized rubber granulates with length of up to 0.5 mm (Figure 1b).

The CRMB was prepared by blending for 30 min the base bitumen with CR particles with a ratio of 20% mass (80% bitumen and 20% CR). A high shear mixer with a speed of 3000 r.p.m. at a temperature of 180 °C was used to prepare the samples. A heating plate and a thermocouple were used to ensure the temperature was maintained throughout the process (180 °C), and the proper digestion of CR was guaranteed [34]. The CRMB1 samples were produced with the CR1 and CRMB2 samples with CR2. The REF samples were produced as the reference bitumen samples without any CR addition. For each mixture, 500 g of CRMB was prepared, and 5 g (±0.01 g) of the mixture was transferred into individual containers with a circular area of 707 mm^2^ (30 mm of inner diameter). The samples were prepared 24 h before the VOC measurements.

### 2.2. VOC Sampling Method

**Figure 2:**
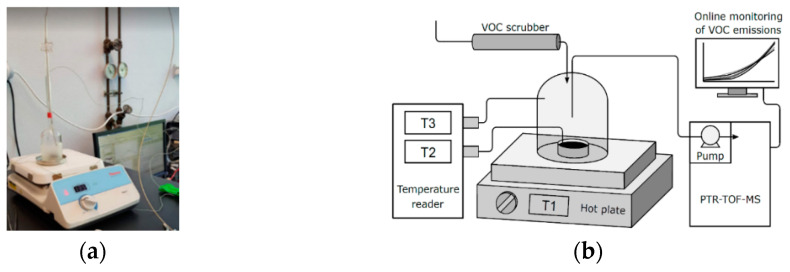
Measurements of volatile organic compounds (VOCs) from a bitumen sample: (**a**) the experimental setup and (**b**) its simplified scheme (T1: hot plate thermometer, T2: thermocouple sensor monitoring bitumen temperature, T3: thermocouple sensor monitoring chamber temperature).

The experimental setup to measure VOCs is shown in Figure 2a. This setup allows heating of the bitumen samples while continuously measuring the fumes emitted at different temperatures. The VOC emission rates are referred to as the units of surface area (nmol m^−2^ s^−1^). Measurements of the background air in the empty chamber were performed to correct the values measured during the tests for a possible influence of the chamber materials and tubing.

The containers with 5 g (±0.01 g) of bitumen were placed in a 120 mL glass chamber on top of a hot plate T1 (Cimarec + HP88857105, ThermoFisher Scientific, Waltham, MA, USA) equipped with a thermostat. A thermocouple sensor monitored the chamber headspace temperature T3, and a second sensor T2 was inserted into the bitumen sample to monitor the actual temperature of the bitumen (see Figure 2b). The fumes were sampled at 100 sccm (0.682 µmol s^−1^) through a 1/16 inch (outside diameter) PEEK (polyether ether ketone) capillary placed 5 cm above the bitumen container. The capillary was directly connected to a PTR-TOF-MS 8000 (Ionicon Analytik GmbH, Innsbruck, Austria) for real-time monitoring of VOC concentrations and further analysis.

The PTR-TOF-MS was operated using H_3_O^+^ as the primary ion. The instrumental conditions of the drift tube were 600 V electric potential, 80 °C temperature, and 2.3 mbar pressure, affording a field density ratio of ≈140 Td (1 Townsend = 10^−17^ cm^2^ V^−1^ s^−1^). A multicomponent gas standard calibration mixture (Apel Riemer, Broomfield, CO, USA) containing 10 VOCs (methanol, acetaldehyde, isoprene, acetone, methyl vinyl ketone, benzene, toluene, t-2-hexen-1-al, c-3-hexen-1-ol, and α-pinene) with masses ranging from m +/z 33 to 137 was used to assess the transmission factor of VOCs of different masses. The gas mixture was diluted through a commercially available gas calibration unit (Ionicon, Innsbruck, Austria) by adding VOC-free air with the same relative humidity as room air. The transmission factors of other compounds were calculated via interpolation using the transmission curve.

The coefficients of reaction between each VOC and H_3_O^+^ were calculated via direct calibration for acetone, benzene, toluene, and hexenol using the abovementioned gas standard calibration. For the other VOCs, approximations were made using values previously described by Cappellin et al. [35], or otherwise, 2 × 10^−9^ cm³ s^−1^ was assumed.

The VOCs identified in the bitumen fumes were grouped into three classes: alkanes, aromatics, and sulfur-containing. The hazard level of each VOC was classified according to the Globally Harmonized System of Classification and Labeling of Chemicals (GHS), which is based on the PubChem database [36].

### 2.3. Procedure to Measure and Identify VOCs

**Figure 3:**
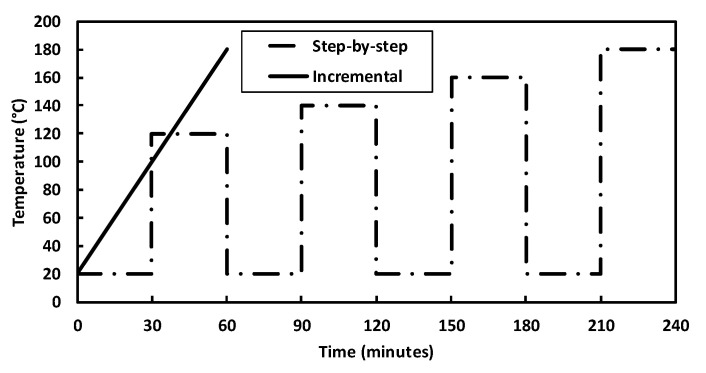
Schematic representation of the heating process for test series 1 (step-by-step) and series 2 (incremental).

To evaluate the influence of temperature on VOC emission rates, six samples (two samples per CR type and two reference samples) were tested. Two series of tests were performed, as shown in Figure 3. The step-by-step experimental procedure was applied in the first series, where the samples were heated in steps of 20 °C from 120 to 180 °C. During each stage, the same sample was first heated to the required temperature (e.g., 120 °C). Then, VOCs were measured, and the sample was cooled until room temperature (20 ± 5 °C). As soon as the sample was cooled enough, it was heated to the next temperature (140 °C), and VOCs were measured again; the same test procedure was repeated for 160 and 180 °C. As two samples of each mixture were used for this series, an average between the two results was used to represent the results.

An incremental increase of temperature was adopted in the second series, where the samples were tested with a continuously increasing temperature from 120 to 180 °C. The VOCs were measured in steps of 10 °C. These two series were adopted in order to evaluate the influence of the heating process on VOC measurements. Because the PTR-TOF-MS is a sensitive equipment, the intention behind the stages in series 1 was to guarantee the temperature was stable during measurements and to avoid interferences from the previous stages on the measurement as fumes from the previous heating might be trapped inside the chamber. In series 2 (with an incremental increase in temperature), the stabilization of temperature and quantity of emission during the test was observed starting from 100 °C. However, the results for analysis were considered starting from 120 °C in order to compare both series.

One specimen of each bitumen (CRMB1, CRMB2, and REF) from the series with the step-by-step increase in temperature was used in the series with an incremental increase in temperature to guarantee that the same mixture and the values from each series could be compared pairwise with each other. Before the start of the second series, each sample was cooled down until room temperature (20 ± 5 °C).

## 3. Results and Discussion

In the current study, a total of 35 VOCs, based on literature, was selected to be measured by the PTR-TOF-MS. These VOCs were emitted by the heated bitumen samples and classified for their hazardous potential according to GHS [36] (see Table 2).

**Figure 4:**
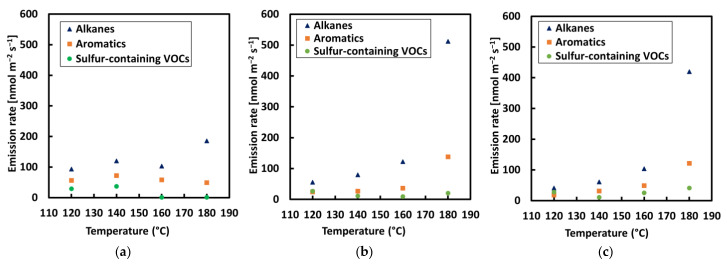
VOC concentration rate of alkanes; aromatics, and sulfur compounds for each sample with a step-by-step increase in temperature: (**a**) REF, (**b**) crumb rubber modified bitumen 1 (CRMB1) and (**c**) CRMB2.

**Figure 5:**
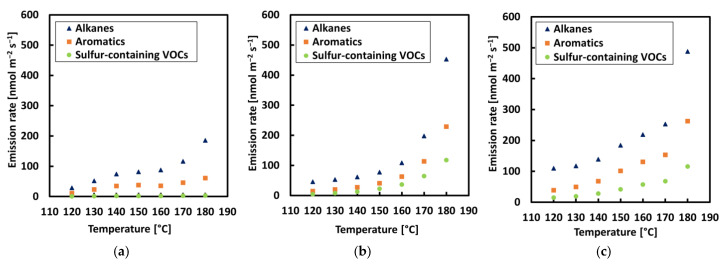
VOC concentration rate of alkanes; aromatics, and sulfur compounds for each sample with an incremental increase in temperature: (**a**) REF, (**b**) CRMB1, and (**c**) CRMB2.

The VOC concentration rate was increased in all bitumen samples with a rise in temperature, with an exponential increase detected between 160 and 180 °C, as can be seen in Figure 4 and Figure 5. The highest rate of VOCs was found in alkanes (lighter VOCs), while the lowest rate was found for sulfur (heavier VOCs). This can be explained by the fact that lighter compounds are released faster; therefore, their emission rate is higher. In general terms, it can be observed that the higher concentration rate for all samples occurred at a temperature of 180 °C in the current study. The temperature is a highly influential factor for bitumen emissions as it is related to molecule stimulation, that is, a higher temperature stimulates molecular movement and, consequently, more compounds escape and are detected by the equipment [2]. The difference in methodology between the two series did not show a significant difference in emission rates.

**Figure 6:**
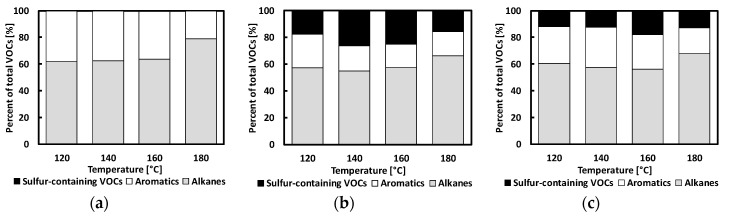
CR influence on the percentage of alkanes, aromatics and sulfur-containing VOC emission with a step-by-step increase in temperature: (**a**) REF, (**b**) CRMB1, and (**c**) CRMB2.

**Figure 7:**
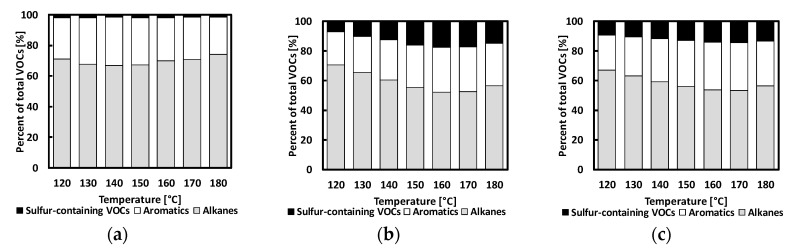
Influence of CR on the percentage of alkanes, aromatics, and sulfur-containing VOC emission with an incremental increase in temperature: (**a**) REF, (**b**) CRMB1, and (**c**) CRMB2.

The addition of CR into bitumen influenced the percentage of alkanes, aromatics, and sulfur released when the mixture was heated in both series of tests (Figure 6 and Figure 7). In these figures, the relative presence of the three VOC classes is displayed in relation to the test temperature. Sulfur compounds were almost nonexistent for base bitumen fumes. A significant increase of sulfur-containing compounds were observed in the fumes for bitumen with CR1 and CR2 addition, even at 120 °C. CRMB1 exhibited more emission of sulfur compounds in comparison to CRMB2. This difference in emission can be explained by the different origins and mechanical/chemical treatment of the CR. The alkanes became more evident in the VOC emissions for bitumen as the temperature increased from 160 to 180 °C. The highest percentage of aromatics was noticed for bitumen within the temperature range of 120–170 °C.

**Figure 8:**
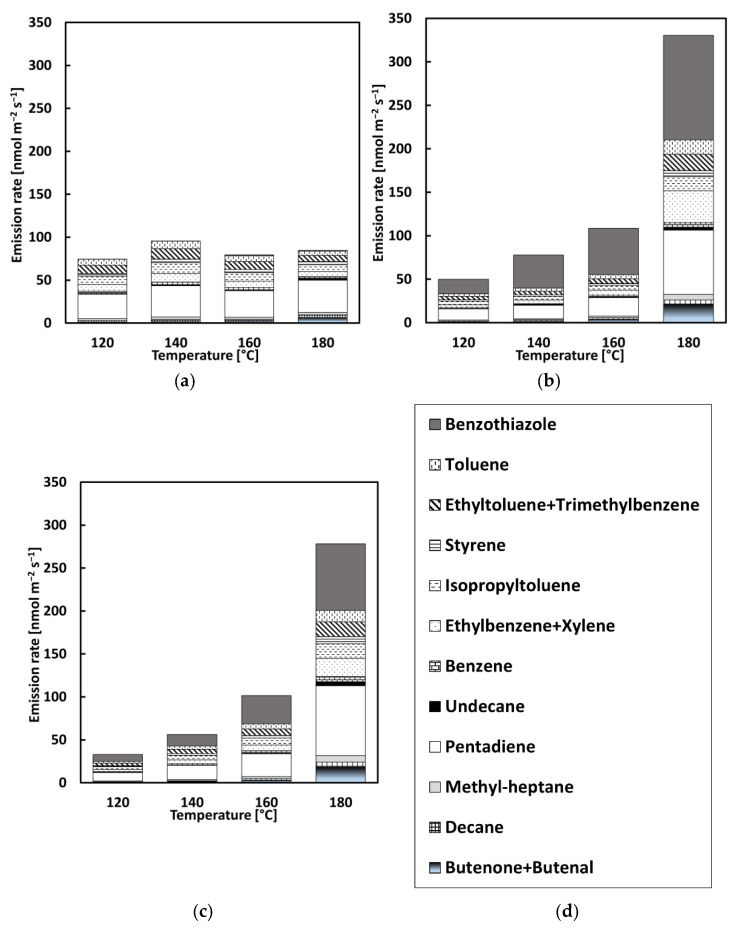
Distribution of VOC emissions with a step-by-step increase in temperature: (**a**) REF, (**b**) CRMB1, (**c**) CRMB2, and (**d**) legend.

**Figure 9:**
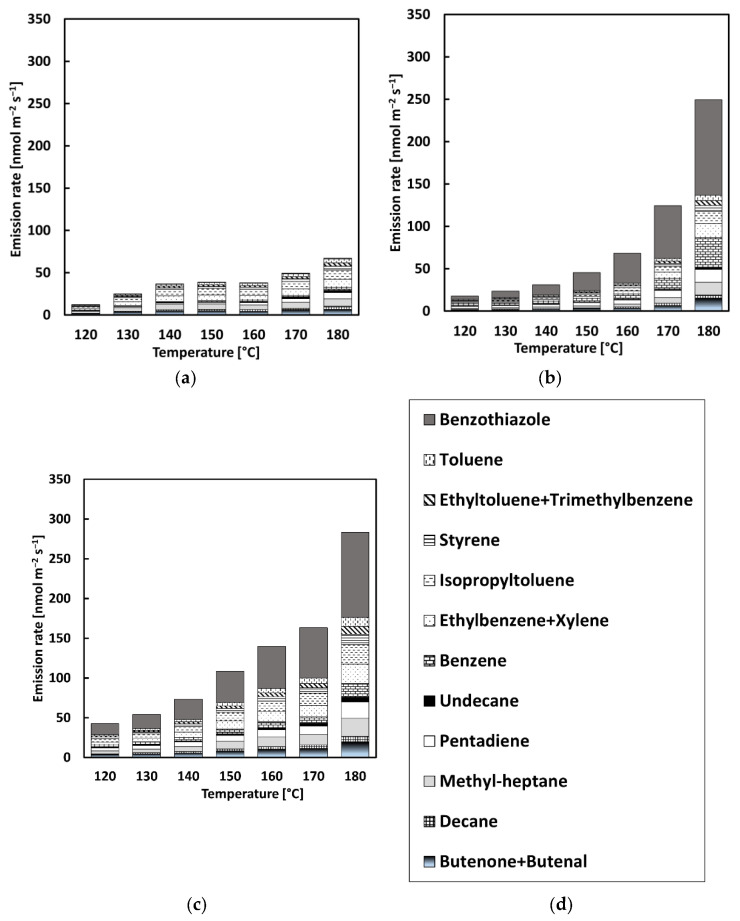
Distribution of VOCs with an incremental increase in temperature: (**a**) REF, (**b**) CRMB1, (**c**) CRMB2, and (**d**) legend.

To evaluate the profile of VOC emissions at different temperatures, 12 VOCs classified as irritant and/or health hazard and/or acute toxic and/or environmental hazard according to the overview shown in Table 2 and with the highest emission rates were chosen, as depicted in Figure 8 and Figure 9. In these figures, the relative presence of the 12 VOC compounds is displayed in relation to the test temperature. CR used for the modification of bitumen can be the source of a variety of hazardous substances released into the environment. This contribution can be clearly noticed for the CRMB1 and CRMB2 samples.

As shown in Figure 8, the analysis confirmed the presence of benzothiazole. The addition of CR in the bituminous binder contributed to the highest emission rate of benzothiazole, which was not observed for the reference bitumen. The concentration of benzothiazole was 65%, 38%, and 35% higher for CR1 in comparison to CR2 at 140, 160, and 180 °C, respectively. The toxicity level values derived for benzothiazole for acute and chronic exposure are intended to eliminate risk factors that impair health (health-protective) [38,39]. Considering the acute, chronic, mutagenic, and carcinogenic effects of benzothiazole, it was included in the assessment of VOCs with risk to human health [38]. The emission of benzothiazole can be detrimental to the health of workers and have an effect on the local flora. Moreover, the use of CR1 in the process of bitumen modification represented a higher emission of benzothiazole compared to CR2. Figure 9 shows that the addition of CR1 and CR2 into bitumen influenced the emission percentage of benzothiazole when the mixtures were heated. In CRMB1 and CRMB2, the highest emission rate of benzothiazole occurred between 170 and 180 °C, the temperature range generally used for the production of hot mix asphalt mixture. In both cases, this emission represented more than 35% of the total emission of compounds classified as irritant, corrosive, health hazardous, or toxic. The increase in temperature resulted in a significant increase in benzothiazole emission due to the digestion process of CR.

In both test series, a clear presence at 180 °C of pentadiene, ethyltoluene + trimethylbenzene, and butanone + butenal was identified for the CR-modified binders. From 160 to 180 °C, the concentration of pendatiene increased by more than 67% for both CRMBs in series 1 and more than 54% in series 2. As for butanone + butenal, an increase of more than 80% was found for both types of CRMBs in series 1 and more than 46% in series 2. The same trend occurred for the other 11 compounds, showing that the increase in temperature from 160 to 180 °C is a crucial factor for VOC emissions.

The analysis in the present study showed a high increase of VOCs, especially benzothiazole, with the addition of CR1 and CR2 to bitumen, potentially leading to more health risks for on-site workers, as confirmed previously in the literature [2,27,37,38,39]. Results confirmed that the 160–180 °C temperature range is a highly influential factor for VOC emissions from heated commercial unmodified bitumen and particularly CRMB. Reducing the temperature of the mixture can decrease the emission of VOCs, especially benzothiazole.

The method introduced in the present study can be implemented as a standardized and reliable method to cross-compare the VOC emission data from different bitumen samples, including with modifications or additives. The characterization of the composition of bitumen fumes by PTR-TOF-MS allows the identification of several VOCs, including those grouped according to the compound classes alkanes, aromatics, and sulfur-containing components. It is also possible to highlight a hierarchy of their appearance in relation to the temperature. The PTR-TOF-MS 8000 achieves a limit of detection of <50 pptv (benzene) at 1 s average and a sensitivity of >100 counts per second per ppbv (benzene) at 40 kHz sampling rate. The extreme sensitivity of this instrument and its sampling time resolution (up to 10 Hz) can be a useful and innovative solution for the asphalt industry to monitor the emissions of VOCs from hot bitumen. Furthermore, it can be coupled to a flux mast to monitor VOC fluxes in situ during asphalt pavement construction—an interesting topic to explore further.

Although the proposed method clearly shows its potential to compare the emissions of samples with and without CR, it is still challenging to correlate these results to the particular circumstances occurring during the production of rubberized asphalt. Further research is needed to establish the relationship between VOC emission concentrations in the laboratory and at road construction sites. Nevertheless, this innovative method can be useful in order to develop and test additives, modification technologies, or methods to control and reduce emissions during the production of crumb rubber modified bitumen.

## 4. Conclusions

VOCs generated on-site during asphalt pavement construction can have a negative effect on the health of workers. The present paper introduces an innovative method to identify VOC emissions for heated bitumen samples under laboratory conditions. This method intends to standardize routine measurements to enable a reliable comparison across bitumen types and their modifications by means of PTR-TOF-MS. The following observations, conclusions, and recommendations are suggested:

1. PTR-TOF-MS can be utilized to analyze VOC emission compounds from heated bitumen samples at temperatures ranging from 120 to 180 °C. Clear differences in VOC emissions were found between commercial unmodified bitumen and bitumen samples after its modification with crumb rubber, which have to be mixed at higher temperatures or in combination with WMA technology.
2. Temperature is a highly influential factor in bitumen VOC emission. A higher temperature stimulates molecular movement and, consequently, lighter compounds (alkanes and aromatics) are released faster. The emission rate exhibits an exponential relationship with temperature increase at the 160–180 °C range.
3. The addition of CR in a bituminous binder contributes to the potentially harmful emission of benzothiazole, which belongs to the sulfur-containing compound class.
4. The CR source affects the amount of VOC emission. The difference in VOCs released in CRMB1 and CRMB2 is due to the different materials and sizes used (CR1 and CR2).
5. The experimental setup developed for this study can be used as a standard method to characterize VOC emissions from different bitumen sources, modifications, or additives as it can be replicated easily and at different temperatures.
6. Very low concentrations of VOCs can be measured due to the extreme sensitivity of PTR-TOF-MS 8000 (>100 cps/ppbv) and the low limit of detection (<50 pptv). Isobaric species can be identified in a split second, and the whole spectrum of VOCs is monitored and recorded in real time.

## Figures and Tables

**Table 1:** Rheological properties of the reference bitumen.

Property	Unit	Results	Test Standard
Penetration (at 25 °C)	dmm	51	EN 1426
Softening point	°C	50.2	EN 1427
Density (at 25 °C)	g/cm^3^	1.025	EN 15326

**Table 2:** List of VOCs identified by proton-transfer reaction time-of-flight mass spectrometer (PTR-TOF-MS) in bitumen samples. The VOCs are classified for their hazardous potential according to the Globally Harmonized System of Classification and Labeling of Chemicals (GHS) [36].

Compound Class	Compound	Molecular Formula	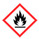	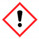	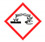	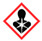	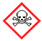	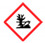	References
			Flammable	Irritant	Corrosive	Health Hazard	Acute Toxic	Environ. Hazard	
Alkanes	Acetic acid	C_2_H_4_O_2_	X	-	X	-	-	-	[2,12]
	Acetone	C_3_H_6_O	X	X	-	-	-	-	[2,37]
	Butenone	C_4_H_6_O	X	X	X	-	X	X	[37]
	Butenal	C_4_H_6_O	X	X	X	-	X	X	[37]
	Cyclohexanone	C_6_H_10_O	X	X	-	-	-	-	[2,37]
	Decane	C_10_H_22_	X	-	-	X	-	-	[2,27]
	Ethylbutenal	C_6_H_10_O	X	X	-	-	X	-	[2,37]
	Hexadienol	C_6_H_10_O	X	X	-	-	-	-	[2,37]
	Heptanal	C_7_H_14_O	X	X	-	-	-	-	[37]
	Hexanal	C_6_H_12_O	X	X	-	-	-	-	[2,37]
	Hexanol	C_6_H_14_O	-	X	-	-	-	-	[2,37]
	Methyl-isobutyl-ketone	C_6_H_12_O	X	X	-	-	-	-	[2,37]
	2-Methyl-furan	C_5_H_6_O	X	X	-	-	X	X	[37]
	Methyl-heptane	C_7_H_16_	X	X	-	X	-	X	[37]
	Methyl-heptyne	C_7_H_12_	X	X	-	X	-	-	[37]
	Pentadiene	C_5_H_8_	X	-	-	-	-	-	[37]
	Pentanal	C_5_H_10_O	X	X	-	-	-	-	[2,37]
	Undecane	C_11_H_24_	-	-	-	X	-	-	[27,37]
Aromatics	Benzaldehyde	C_7_H_6_O	-	X	-	-	-	-	[37]
	Benzene	C_6_H_6_	X	X	-	X	-	-	[12,37]
	Bromo-benzene	C_6_H_5_Br	X	X	-	-	-	X	[12]
	Butyl-benzene	C_10_H_14_	X	X	-	-	-	X	[12]
	Diethylthiophene	C_8_H_12_S	-	X	-	-	-	-	[2]
	Ethylbenzene	C_8_H_10_	X	X	-	X	-	-	[2,12,37]
	Xylene	C_8_H_10_	X	X	-	X	-	-	[2,12,37]
	Ethylphenyl-ethanone	C_10_H_12_O	-	X	-	-	-	-	[37]
	Ethyltoluene	C_9_H_12_	X	X	-	X	-	X	[12]
	Trimethylbenzene	C_9_H_12_	X	X	-	-	-	-	[12]
	Isopropyltoluene	C_10_H_14_	X	-	-	X	-	X	[12]
	Methyl-benzaldehyde	C_7_H_6_O	-	X	-	-	-	-	[37]
	Styrene	C_8_H_8_	X	X	-	X	-	-	[12]
	Toluene	C_7_H_8_	X	X	-	X	-	-	[2,12,37]
	Trichloro-benzene	C_6_H_3_Cl_3_	-	X	-	-	-	X	[12]
Sulfur	Benzothiazole	C_7_H_5_NS	-	X	-	-	X	-	[2,27,37,38,39,40,41]
	Sulfur-diozide	S_8_	-	X	-	-	-	-	[37]
