# Correction: Borinelli et al. VOC Emission Analysis of Bitumen Using Proton-Transfer Reaction Time-Of-Flight Mass Spectrometry. *Materials* 2020, *13*, 3659

**DOI:** 10.3390/ma15103506

**Published:** 2022-05-13

**Authors:** Jaffer Bressan Borinelli, Johan Blom, Miguel Portillo-Estrada, Patricia Kara De Maeijer, Wim Van den bergh, Cedric Vuye

**Affiliations:** 1 Road Engineering Research Section (RERS), EMIB, Faculty of Applied Engineering, University of Antwerp, 2020 Antwerp, Belgium; johan.blom@uantwerpen.be (J.B.); wim.vandenbergh@uantwerpen.be (W.V.d.b.); cedric.vuye@uantwerpen.be (C.V.); 2 Research Group PLECO (Plants and Ecosystems), Faculty of Science, University of Antwerp, 2610 Wilrijk, Belgium; miguel.portilloestrada@uantwerpen.be; 3 Built Environment Assessing Sustainability (BEASt), EMIB, Faculty of Applied Engineering, University of Antwerp, 2020 Antwerp, Belgium; patricija.karademaeijer@uantwerpen.be

The authors wish to make the following corrections to this paper [1]:

## Text Correction

There was an error in the original publication. The values of emission should be expressed as nmol m^−2^ s^−1^ (nanomole m^−2^ s^−1^), instead of µmol m^−2^ s^−1^ (micromole per square meter and second). A correction has been made to: 2. Materials and Methods, 2.2. VOC Sampling Method.

The VOC emission rates are referred to as the units of surface area (nmol m^−2^ s^−1^).

## Error in Figure

In the original publication, there was a mistake in Figure 4, Figure 5, Figure 8 and Figure 9. After publication and continuing with our line of research, the authors have pointed out a mistake in the y-axis of the Figure 4, Figure 5, Figure 8 and Figure 9. In the published version, the values of emission should be expressed as nmol m^−2^ s^−1^ (nanomole m^−2^ s^−1^), instead of µmol m^−2^ s^−1^ (micromole per square meter and second). The corrected figures appear below. The authors apologize for any inconvenience caused and state that the scientific conclusions are unaffected. This correction was approved by the Academic Editor. The original publication has also been updated. 

## Figures and Tables

**Figure 4:**
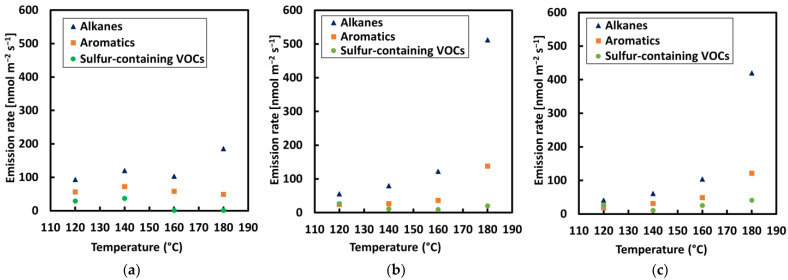
VOC concentration rate of alkanes; aromatics, and sulfur compounds for each sample with a step-by-step increase in temperature: (**a**) REF, (**b**) crumb rubber modified bitumen 1 (CRMB1), and (**c**) CRMB2.

**Figure 5:**
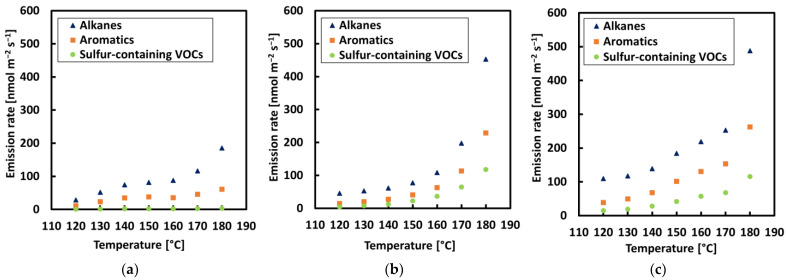
VOC concentration rate of alkanes; aromatics, and sulfur compounds for each sample with an incremental increase in temperature: (**a**) REF, (**b**) CRMB1, and (**c**) CRMB2.

**Figure 8:**
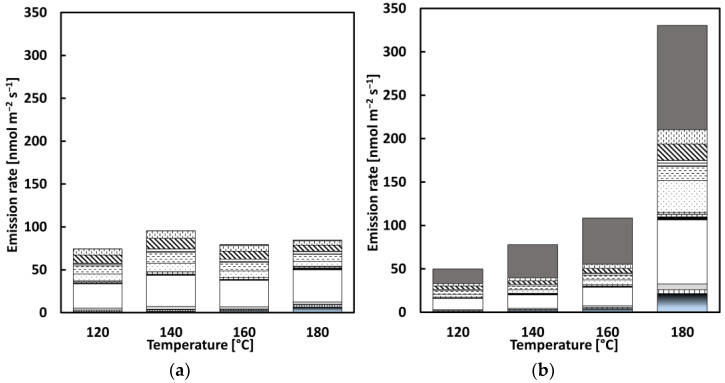

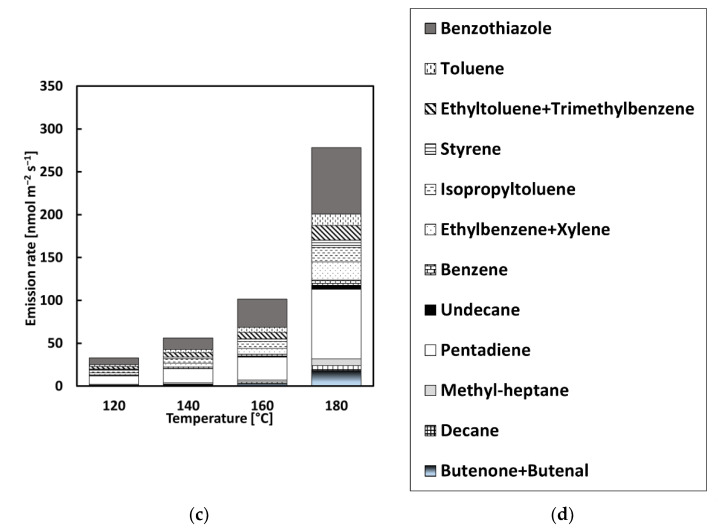
Distribution of VOC emissions with a step-by-step increase in temperature: (**a**) REF, (**b**) CRMB1, (**c**) CRMB2, and (**d**) legend.

**Figure 9:**
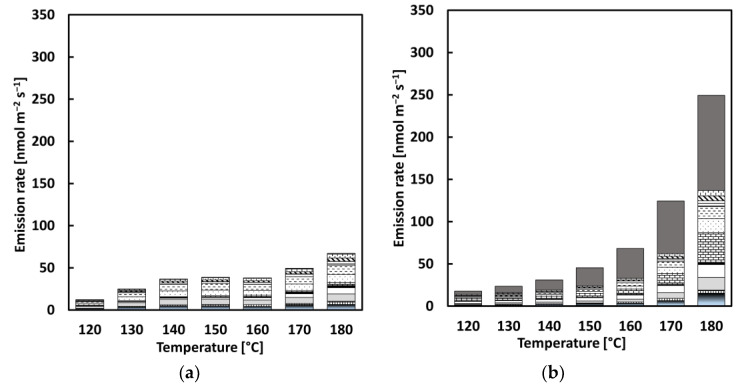

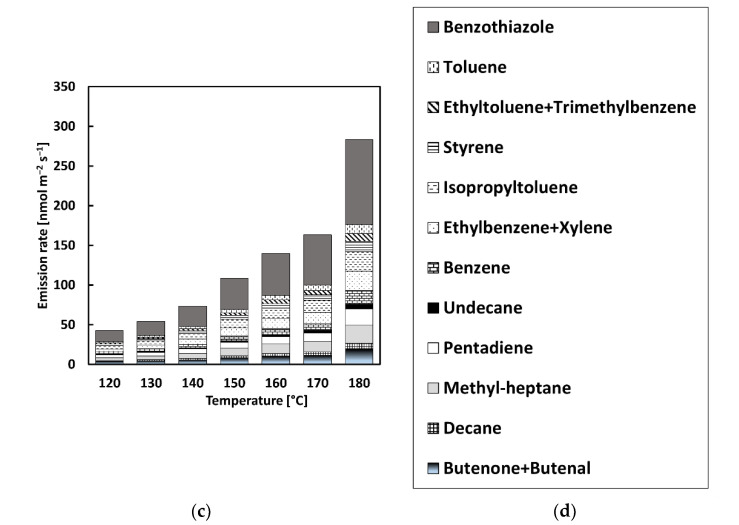
Distribution of VOCs with an incremental increase in temperature: (**a**) REF, (**b**) CRMB1, (**c**) CRMB2, and (**d**) legend.
